# Supplementary material for: Metformin Inhibits Tumor Metastasis through Suppressing Hsp90α Secretion in an AMPKα1-PKCγ Dependent Manner
Source: Cells. 2020 Jan 7;9(1):144. doi: 10.3390/cells9010144 (PMC7016760; doi:10.3390/cells9010144)
Supplement: Supplementary file 1 [file cells-09-00144-s001.pdf]

Supplementary Material

Metformin Inhibits Tumor Metastasis through Suppressing Hsp90α Secretion in an AMPKα1-PKCγ Dependent Manner

Yuanchao Gong [1,2,3], Caihong Wang [1,2,3], Yi Jiang [1,2,3], Shaosen Zhang [1,2,3], Shi Feng [1,2,3], Yan Fu [1,2,3] and Yongzhang Luo [1,2,3,*]

1 The National Engineering Laboratory for Anti-Tumor Protein Therapeutics, Tsinghua University, Beijing 100084; China; gongyc14@mails.tsinghua.edu.cn (Y.G.); wangch15@mails.tsinghua.edu.cn (W.C.); jiang-y17@mails.tsinghua.edu.cn (Y.J.); zhangss14@mails.tsinghua.edu.cn (S.Z.); fengs14@mails.tsinghua.edu.cn (S.F.); fuyan@tsinghua.edu.cn (Y.F.)

2 Beijing Key Laboratory for Protein Therapeutics, Tsinghua University, Beijing 100084; China

3 Cancer Biology Laboratory, School of Life Sciences, Tsinghua University, Beijing 100084, China

* Correspondence: yluo@mail.tsinghua.edu.cn; Tel.: +86-10-6277-2897; Fax: 86-10-6279-4691

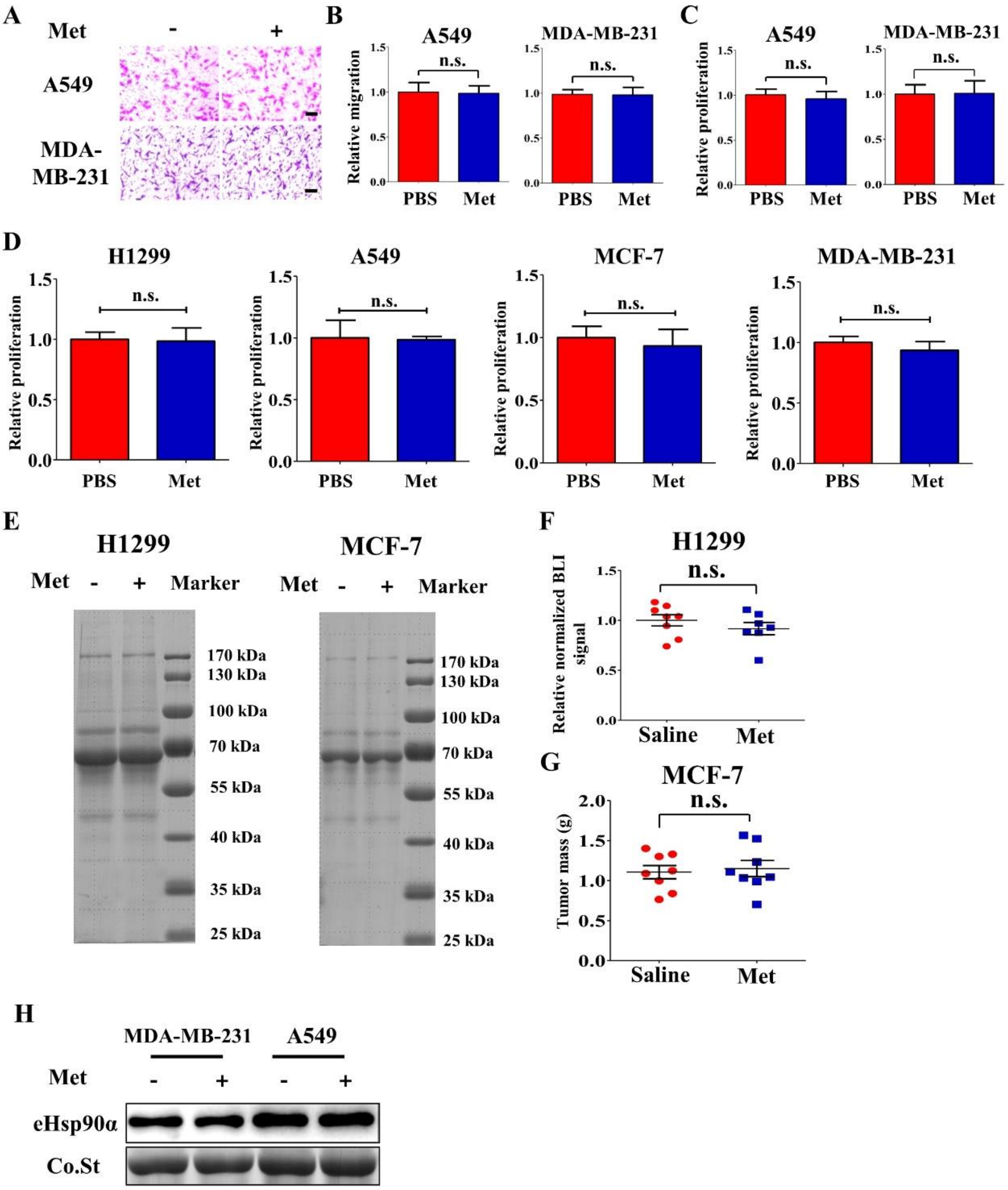

Figure S1. Metformin has no effect on proliferation but inhibits Hsp90α secretin. (A) Representative images and (B) quantified results of A549 and MDA-MB-231 cells migration assay treated with PBS or metformin (200 μM). Scale bar, 100 μm. **p$<$0.01, ***p$<$0.001. The effects of metformin (200μM) on A549 and MDA-MB-231 cells proliferation in vitro at 48h (C) and 24h (D). Cells were seeded into 96-well plates and cell proliferation was examined by CCK-8 assays. (E) Conditioned medium derived from H1299 and MCF-7 cells were subjected to SDS-PAGE before mass spectrometry. (F) Quantified results of H1299 primary tumor in representative bioluminescent (BLI) images. (G) Tumor mass of MCF-7 cells in nude mice. (H) The conditioned medium (CM) of MDA-MB-231 and A549 cells was collected and concentrated, and then extracellular Hsp90α (eHsp90α) was measured by Western blot. Co.St (Coomassie brilliant blue) was used as a control.

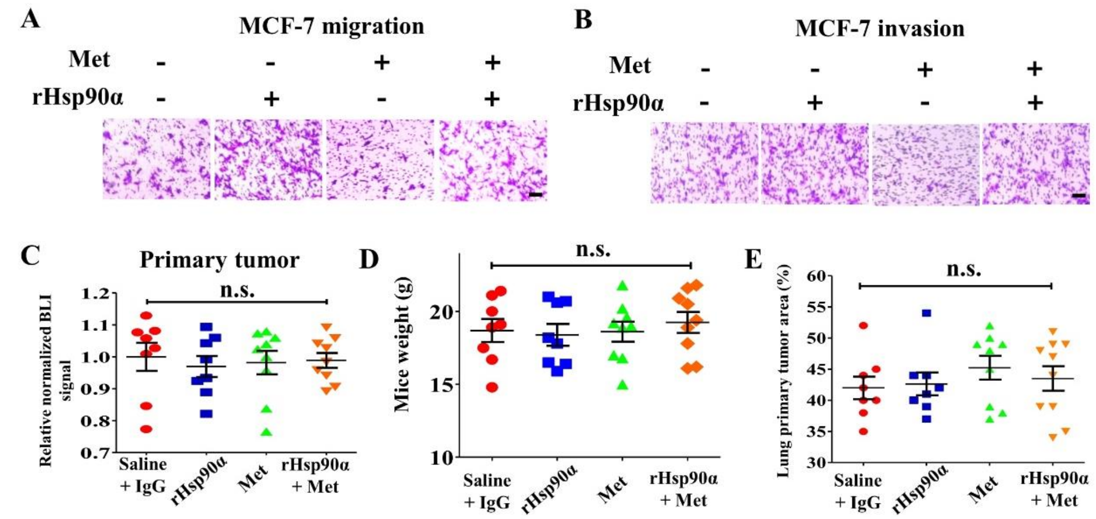

Figure S2. Metformin has no effect on tumor growth *in vivo*. Representative images of cell migration (A) and invasion (B) in MCF-7 cells treated with or without metformin (200 μM) and recombinant Hsp90α (10 ng/mL). Scale bar, 100 μm. (C) Quantified results of H1299 primary tumor in representative bioluminescent images (BLI). (D) The weight of mice injected with H1299 cells treated with or without metformin and recombinant Hsp90α. (E) Quantified results of primary tumor in H&E staining.

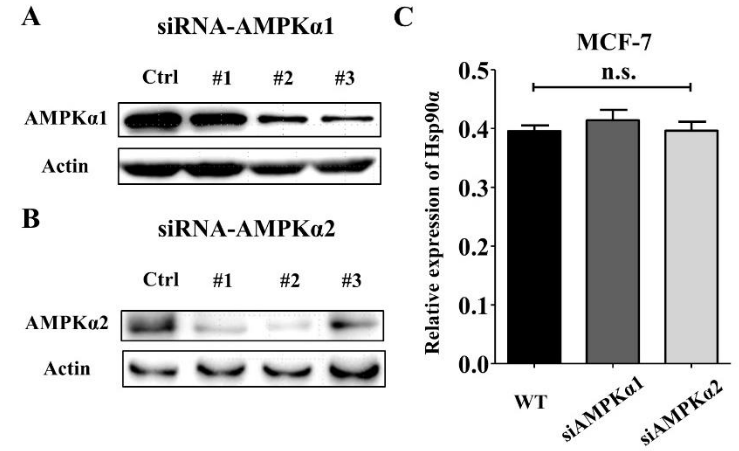

Figure S3. The knockdown efficiency of siRNAs. The efficiency of siRNAs for AMPKα1 (A) and AMPKα2 (B) knockdown was detected by using Western blots. (C) The effects of AMPKα1 and AMPKα2 on proliferation were examined by CCK8 assay in MCF-7 cells.

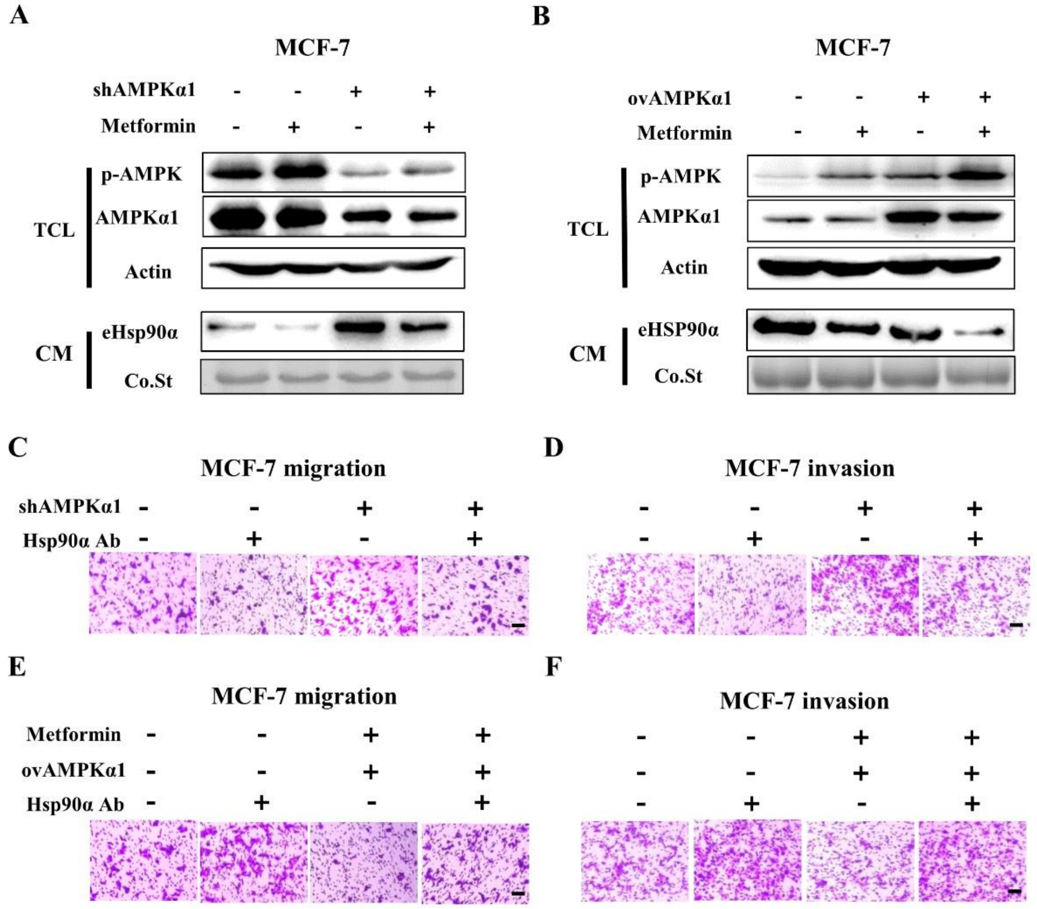

Figure S4. Metformin inhibits Hsp90α secretion dependent on AMPKα1. Extracellular Hsp90α was measured in AMPKα1 KD (A) and AMPKα1 OV (B) MCF-7 cells treated with or without metformin (200 μM). Western blots of AMPKα, p-AMPK and actin were also shown. Representative images of MCF-7 KD cell migration (C) and MCF-7 cell invasion (D) treated with or without Hsp90α antibody. Representative images of MCF-7 OV cell migration (E) and MCF-7 cell invasion (F) treated with or without recombinant Hsp90α and metformin. Scale bar, 100 μm.

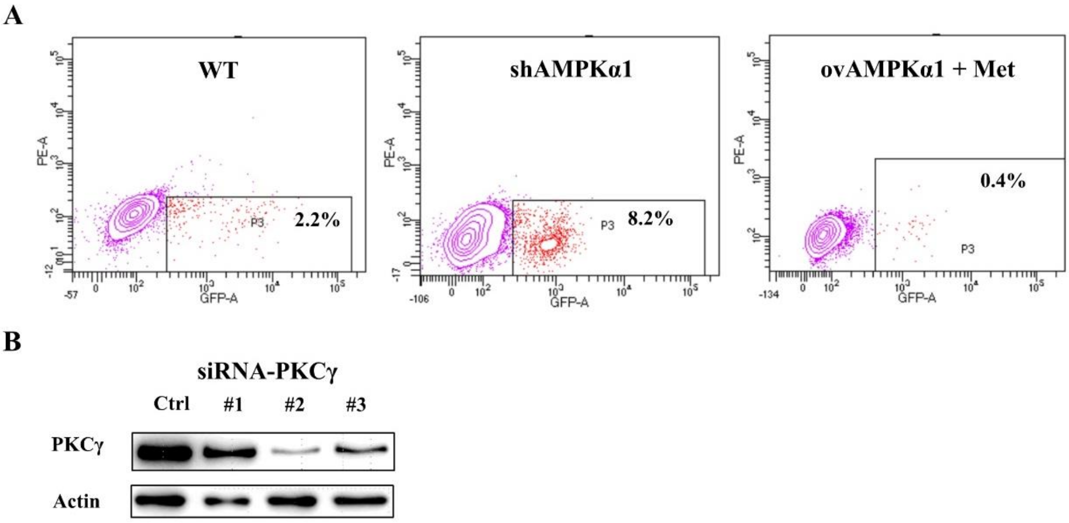

Figure S5. AMPKα1 inhibits the membrane translocation of Hsp90α. (A) Hsp90α on the cell membrane was measured by flow cytometry in MCF-7-WT, KD and OV cells. (B) The efficiency of siRNAs for PKCγ was detected by using western blots.

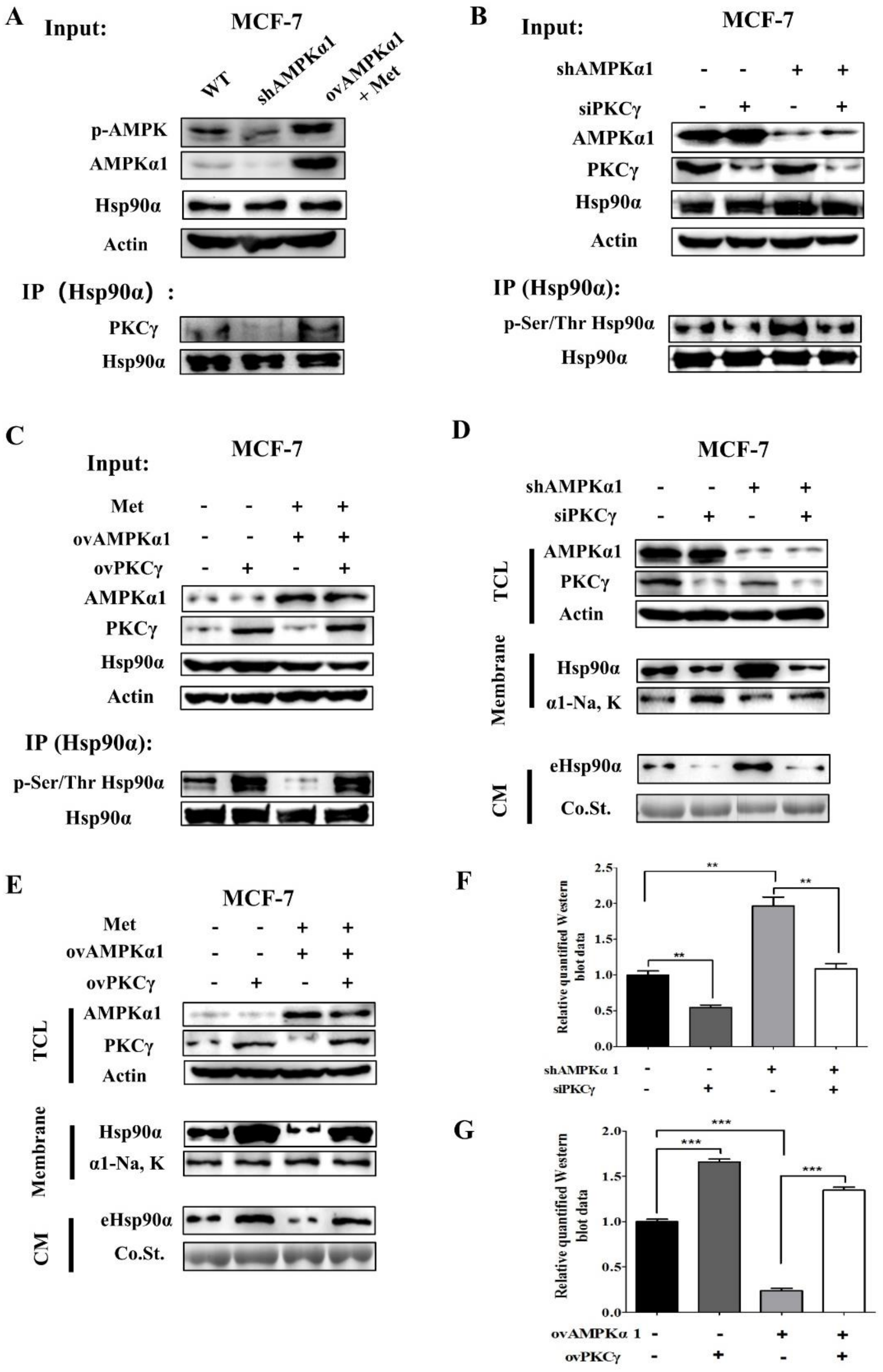

Figure S6. AMPKα1 inhibits Hsp90α phosphorylation, membrane translocation and secretion by suppressing the kinase activity of PKCγ. (A) Hsp90α was pulled down in MCF-7-WT, KD and OV cells treated with or without metformin. PKCγ was measured by western blot. (B) Hsp90α was pulled down in WT, AMPKα1-KD, PKCγ-KD and AMPKα1-PKCγ-double KD MCF-7 cells. The phosphorylation level of Hsp90α at Ser/Thr was measured by Western blot. (C) Hsp90α was pulled down in WT, AMPKα1-OV, PKCγ-OV and AMPKα1-PKCγ-double OV MCF-7 cells treated with or without metformin. The phosphorylation level of Hsp90α at Ser/Thr was measured by western blot. (D) Plasma membrane extractions and conditioned medium of WT, AMPKα1-KD, PKCγ-KD and AMPKα1-PKCγ-double KD MCF-7 cells were analyzed by western blot. Na, K-ATPase α1 was the plasma membrane marker. (E) Plasma membrane extractions and conditioned medium of WT, AMPKα1-OV, PKCγ-OV and AMPKα1-PKCγ-double OV MCF-7 cells treated with or without metformin were analyzed by western blot. Na, K-ATPase α1 was the plasma membrane marker. The quantified phosphorylation level of Hsp90α in Figure 6D and 6E.

Table S1. siRNA sequences for knock down experiments

Target Gene	Sense (5'-3')	Antisense (5'-3')
siAMPKα1 #1	GCGUGUACGAAGGAAGAAUTT	AUUCUUCCUUCGUACACGCTT
siAMPKα1 #2	GAGGAGAGCUAUUUGAUUATT	UAAUCAAAUAGCUCUCCUCTT
siAMPKα1 #3	CGGGAUCAGUUAGCAACUATT	UAGUUGCUAACUGAUCCCGTT
siAMPKα2 #1	GGCUCUUUCAGCAGAUUCUTT	AGAAUCUGCUGAAAGAGCCTT
siAMPKα2 #2	CCACUCUCCUGAUGCAUAUTT	AUAUGCAUCAGGAGAGUGGTT
siAMPKα2 #3	CAGGUCCUGAAGUUGAUAUTT	AUAUCAACUUCAGGACCUGTT
siPKCγ #1	GCAGAUGAGAUCCACGUAATT	UUACGUGGAUCUCAUCUGCTT
siPKCγ #2	GCCUCUUCUUCCUUCACAATT	UUGUGAAGGAAGAAGAGGCTT
siPKCγ #3	CCGACUUCAGCUUCCUCAUTT	AUGAGGAAGCUGAAGUCGGTT

Table S2. Primer sequences for PCR analysis

Target Gene	Experiment	Forward	Reverse
AMPKα1	PCR	GCATGCGCAGACTCAGTTCC	CGTTATTGTGCAAGAATTTTAATTAG
AMPKα2	PCR	GCATGGCTGAGAAGCAGAAG	CGTCAACGGGCTAAAGTAG
PKCγ	PCR	GCTCTAGAATGGCTGGTCTGGGC CCCGGC	CGACGCGTTTACATGACGGGCAC

Table S3. The information of antibodies used in this study

Antibody	Host species	Supplier	Catalog number	Dilution
AMPKα1	Rabbit	Cell Signaling Technology	2795T	1:1000
AMPKα2	Rabbit	Cell Signaling Technology	2757T	1:1000
Hsp90α	Mouse	Protgen	D10	1:1000
Phospho-AMPK	Rabbit	Cell Signaling Technology	2535T	1:1000
PKCγ	Rabbit	Cell Signaling Technology	59090S	1:1000
Phospho-PKCγ	Rabbit	Affinity	AF8347	1:1000
Phospho-(Ser/Thr)	Rabbit	Abcam	Ab117253	1:1000
α1-Na, K	Rabbit	Cell Signaling Technology	3010S	1:1000
